# Estimating reef fish size distributions with a mini remotely operated vehicle-integrated stereo camera system

**DOI:** 10.1371/journal.pone.0247985

**Published:** 2021-03-04

**Authors:** Steven B. Garner, Aaron M. Olsen, Ryan Caillouet, Matthew D. Campbell, William F. Patterson

**Affiliations:** 1 Fisheries and Aquatic Sciences, University of Florida, Gainesville, Florida, United States of America; 2 Department of Ecology and Evolutionary Biology, Brown University, Providence, Rhode Island, United States of America; 3 Mississippi Laboratories, Southeast Fisheries Science Center, National Marine Fisheries Service, Pascagoula, Mississippi, United States of America; California Academy of Sciences, UNITED STATES

## Abstract

We tested the efficacy of a stereo camera (SC) system adapted for use with a remotely operated vehicle (ROV) to estimate fish length distributions at reef sites in the northern Gulf of Mexico. A pool experiment was conducted to test the effect of distance (1, 2, 3 or 5 m), angle of incidence (AOI; 0° to 40° at 5° increments), and SC baseline distance (BD; BD1 = 406, BD2 = 610, and BD3 = 762 mm camera separation) on the accuracy and precision of fish model length (288, 552, or 890 mm fork length) estimates compared to a red laser scaler (RLS). A field experiment was then conducted at 20 reef sites with SCs positioned at BD1 to compare fish length distribution estimates between the SC and RLS systems under *in situ* conditions. In the pool experiment, mean percent errors were consistently within the *a priori* selected threshold of ±5% at AOIs ≤10° at all distances with all four systems. However, SCs produced accurate estimates at AOIs up to 30° at all distances tested; 2–3 m was optimal. During reef site surveys, SCs collected 10.4 times as many length estimates from 4.3 times as many species compared to the RLS. Study results demonstrate that, compared to laser scalers, ROV-based SC systems can substantially increase the number of available fish length estimates by producing accurate length estimates at a wider range of target orientations while also enabling measurements from a greater portion of the cameras’ field of view.

## Introduction

Fish length data are commonly used to examine ecological processes [1–4] or assess the status of populations [5, 6] because length is strongly correlated with various life-history parameters [7]. Thus, size-composition data can provide critical demographic information in a variety of ecological modeling and stock assessment contexts including evaluations of predator-prey relationships [8–10], ontogenetic shifts in habitat use [11–13], sustainable harvest levels [14–16], or ecosystem-level effects [17–19]. Video-based methods for estimating length distributions can reduce sampling bias due to gear selectivity and provide an ethical improvement over traditional sampling methods when incidental mortality is common [20, 21]. Visually derived length estimates are particularly valuable when even minor handling-induced mortality is a concern (e.g., endangered species) or when fish reside in protected areas and are thus unavailable for collection [22].

Methods for collecting fish length data with stereo cameras (i.e., photogrammetry) developed rapidly in the 1980s and have since been adapted for a wide variety of scientific needs, including use in reef fish community surveys conducted with divers or remotely operated vehicles (ROVs) [23–28]. To collect viable length estimates with stereo cameras, paired cameras are fixed to a survey gear and positioned so that fields-of-view overlap; their respective orientations are then calibrated [23, 29–31]. Length estimates can be collected for a wide variety of objects in the environment, provided objects are viewed simultaneously by both cameras [28–30], which may increase sample size and accuracy compared to traditional methods that utilize visually estimated size classes [32–35] or laser scalers [36–39]. Compared to other common survey gears, ROVs can avoid duration limits, descend to deeper depths, and minimize fish attraction or avoidance behaviors associated with divers while also allowing density estimates unavailable with stationary camera systems.

Stereo-camera methods have recently been adapted to both working-class [27] and mini-class ROVs [28] and length measurements collected via commercially available software. However, Olsen and Westneat [40] recently developed an efficient stereo camera calibration and measurement software (StereoMorph [41]) freely available as a package within the R environment [42]. The goal of this study was to assess the efficacy of collecting robust length estimates for reef fish communities via small, low-cost (<$500), hand-held action cameras in stereo integrated with a (micro) mini-class ROV (<10 kg; hereafter referred to simply as ROV) using the newly developed freeware. This was accomplished by first conducting a pool experiment to test the effects of distance, angle-of-incidence, and SC baseline distance (i.e., inter-camera distance, BD) on length estimates for fish models of known length compared to a traditional red laser scaler (RLS). Based on the results from the pool experiment, a single SC pair with optimal BD was selected for field trials by integrating it with the ROV that is also equipped with a red laser scaler. Field trials were then conducted to estimate fish length distributions at northern Gulf of Mexico reef sites to compare the amount and quality of length composition data produced by either system.

## Materials and methods

No permits were necessary to conduct the study because field sites were not on privately owned land and no animals were collected.

### Pool experiment

**Fig 1:**
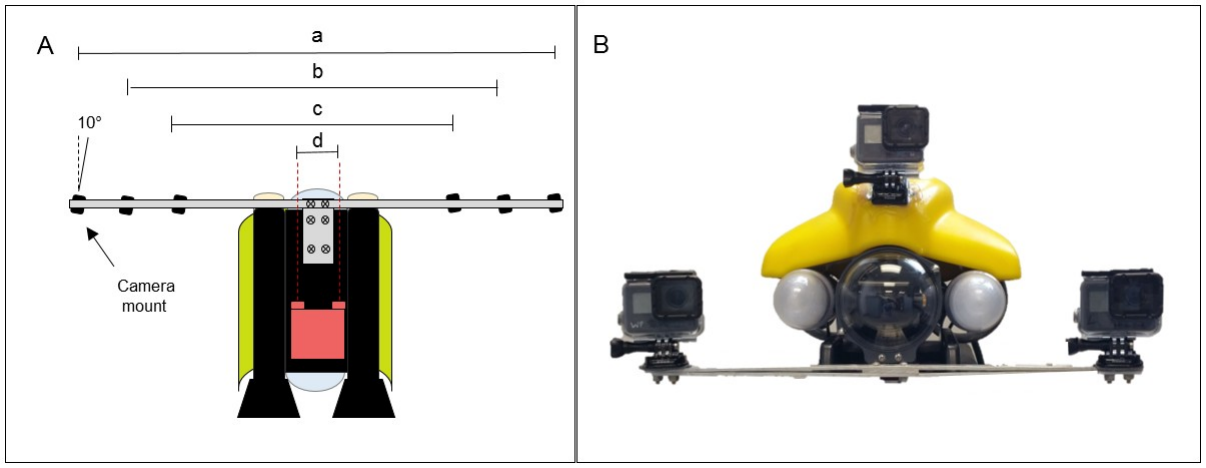
Stereo camera attachment and orientation. Schematic showing A) bottom view of the three stereo camera pairs mounted to the ROV at BDs of a) 762 mm (BD3), b) 610 mm (BD2), or c) 406 mm (BD1) and the d) RLS (75 mm BD) used to estimate model fish lengths in the pool experiment. Panel B indicates the front-view of the digital cameras (GoPro Hero5) mounted to the ROV in stereo at BD1 for use in the field experiment. Black rectangles in panel A indicate the mounting positions of the six GoPro Hero5 model cameras (3 pairs), each of which had 10° inward rotation.

**Fig 2:**
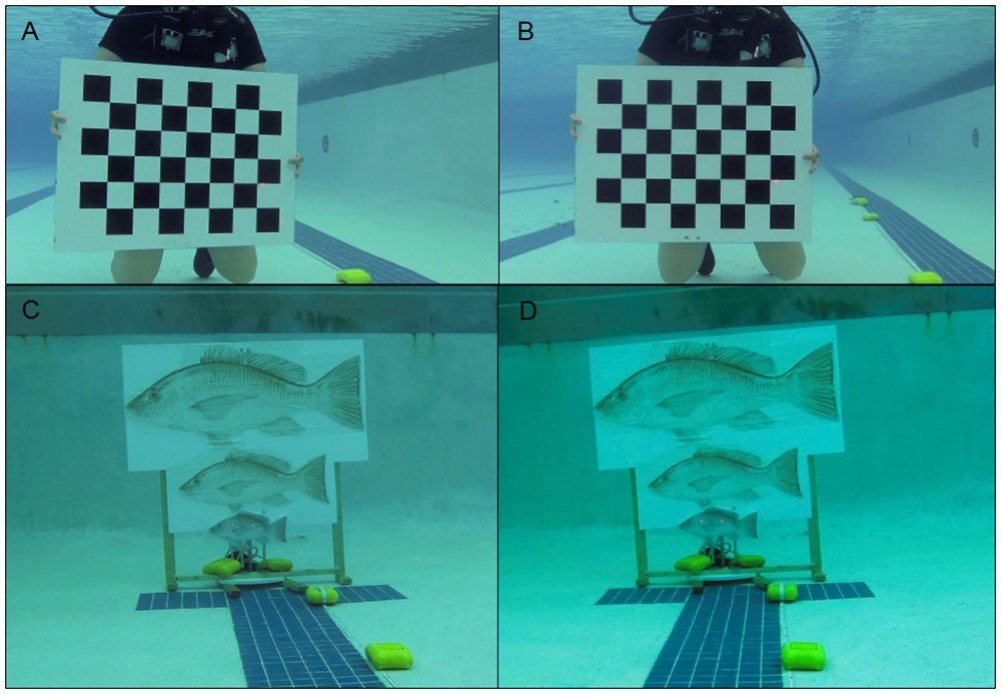
Paired images of calibration checkerboard and fish models. Example paired images from A) left or B) right view of calibration checkerboard (457 x 610 mm; 63.7 mm square size; 2 m distance) and C) left or D) right stereo camera view of fish display board indicating small (288 mm FL), medium (552 mm FL), or large (890 mm FL) paper red snapper models viewed at 3 m distance and perpendicular to the ROV. Laser points are visible on the smallest red snapper model in panels C and D.

Underwater video was collected with high-definition GoPro Hero5 digital cameras (n = 3 pairs) that were enclosed in standard submersible GoPro camera housings (60 m depth rating) and mounted to a VideoRay Pro4 ROV (375 x 289 x 223 mm; 6.1 kg; 305 m depth rating) equipped with an RLS (2 parallel 5 mw 635 nm Class IIIa red lasers, 75 mm BD). All three camera pairs were mounted to a single aluminum bar (800 x 38 x 6 mm) via an adhesive mounting pad with two stainless-steel through-bolts and lock nuts. The aluminum bar with cameras was attached perpendicularly at the midpoint to a 76 x 6 mm flat aluminum plate via three stainless-steel through-bolts and lock nuts to form a T-shaped bar. The T-bar was then mounted to the ROV via manufacturer-drilled, threaded mounting holes on the underside of the ROV’s sled. Camera pairs were mounted to the aluminum bar at BDs of 406 (BD1), 610 (BD2), or 762 mm (BD3), with the anterior-posterior axis of the ROV bisecting each SC pair (Fig 1). Each camera case was mounted inward 10° (toe-in angle) toward the center line of the ROV and each camera was set to the narrow field-of-view (FOV; 49.1° vertical and 64.6° horizontal, 28 mm focal length equivalent) at 1080p definition with a 60-fps frame rate. A black and white checkerboard printed on vinyl and mounted to a 610 x 457 mm Lexan polycarbonate sheet was used to calibrate all SCs. Each square of the checkerboard measured 63.7 x 63.7 mm, with a total of 7 horizontal and 5 vertical inner corners (Fig 2A and 2B). Immediately after initializing recording on each of the six cameras, a flashlight was triggered to allow post-processing synchronization between video cameras for extracting paired images. Following the methods of Delacy et al. [43], the checkerboard was positioned throughout the FOV by forming expanding concentric circles in a clockwise pattern at AOIs from 0 to 20° from perpendicular at distances of 1, 2, 3, and 5 m from the ROV. Paired images used for calibration were extracted at a representative number (n = 50) of AOI and distance combinations throughout the FOV. Checkerboard image pairs were taken simultaneously from all three SC pairs to minimize the potential effect of image pair selection on measurement errors. Three replicate trials were conducted to account for differences in calibration quality.

Following completion of the calibration video, red snapper, *Lutjanus campechanus*, models of known length (288, 552, or 890 mm fork length, FL), were submerged in the pool and simultaneously filmed by each SC pair. Fish models were two-dimensional images printed on waterproof paper and adhered to a polyvinyl chloride (PVC) board affixed to a weighted wooden stand (Fig 2C and 2D). A circular disk was demarcated from 0 to 40° at 5° increments and mounted to a metal rod affixed to the back of the PVC board, which enabled the entire stand to be precisely positioned at each AOI (0, 5, 10, 15, 20, 25, 30, 35, or 40° from perpendicular). A transect tape was used to position the ROV at each designated distance (1, 2, 3, or 5 m) from the PVC board, and tile grids built into the floor of the pool allowed for perpendicular positioning of the ROV throughout the experiment. For each model distance and AOI, the ROV was positioned at a level height above the pool floor such that the RLS was visible along the lateral midline of each fish model (Fig 2C and 2D). Thus, up to 9 measurements were available for each fish model from each SC pair at each model distance and AOI combination from all three trials. However, all three models were not visible in every image at 1 m distance due to either the narrow FOV setting or the AOI.

Calibration and measurement videos were synchronized and still images extracted with CyberLink’s PowerDirector 15 video processing software. Camera calibration parameters and model length estimates were estimated with the StereoMorph package [40, 41] in R [42]. The StereoMorph package identifies common points (i.e., internal corners for checkerboard squares of known dimension) among paired images of checkerboard positions to estimate image distortion parameters and 6 optimal transformation parameters (3 translational and 3 rotational) to sequentially relate each set of checkerboard image pairs in 3-dimensional space based on minimizing calibration error. The transformation parameters are then used to estimate calibration coefficients (by direct linear transformation) to transform 2-dimensional image coordinates into 3-dimensional coordinates for collecting measurements [40, 41]. Each image pair was digitized and two landmarks were identified for each target: the anterior-most point of the premaxilla and the posterior-most point of the caudal fin at the midline (i.e., the tail fork). The distance between these two landmarks comprised the fork length (FL) estimate used for analyses.

Single still images used to estimate model lengths with the RLS were taken from an additional, forward-facing GoPro mounted atop the ROV at 0° tilt and 0° toe-in-angle (Fig 1B). Length estimates were generated for the RLS by dividing model fork length by laser inter-point distance as measured on screen when striking each model. This ratio was then multiplied by the RLS BD (75 mm) to generate each model length estimate. As laser position is fixed in parallel and calibration quality was not a concern with the RLS, length measurements were estimated with the RLS during only one trial producing up to 3 measurements for each model at each distance and AOI combination. As with the SCs, all three models were not always visible in every image at 1 m distance due to either the narrow FOV setting or the AOI.

Bias in each fish FL estimate was calculated as percent error (PE) with the equation:

$$PE_{ij}=(\frac{\left(estimated\;length_{ij}-actual\;length_{ij}\right)}{actual\;length_{ij}})*100$$ (1)

where estimated length is the length estimate derived for each fish model *i* from each measurement system *j*. Percent error was used to identify measurement bias, with the threshold accuracy set *a priori* to ±5%. However, the absolute value of the percent error estimate (absolute percent error, APE) was calculated as the response variable for comparing differences in bias among SC pairs and the RLS. The effect of each factor, and their interactions, on APE was tested in a generalized linear model (GLM) framework in R [42] by specifying a Gamma distribution with logistic (i.e., log-link) link function between the independent factors and the response variable (i.e., x+1 transformed APEs). The dispersion parameter was set equal to 1 (i.e., an exponential distribution) because the APE data were always positive with multiplicative errors. The factor effects modeled were the measurement system (4 levels: SC BD1, BD2, or BD3, or RLS), model FL (3 levels: 288, 552, or 890 mm FL), distance (m), and AOI (degrees) at an *a priori* significance level of α = 0.05. The relative position (3 levels: centered or above or below midline) of each model in the camera’s view was included as a covariate to test the effect of distortions or reduced calibration accuracy as models were measured farther from the center of view. Centered referred to the model at the center of view in each still image as indicated by laser points. For example, if lasers were visible at the midline of the small fish model, then the medium model (one position above center) would be classified as 1 and the large model would be given a classification of 2; with lasers present at the midline of the middle fish model, both small and large models would be classified as 1.

### Stereo camera field trials

Fish communities were surveyed with an ROV integrated with both a SC and RLS system at 20 northern Gulf of Mexico (nGOM) reef sites to test the efficacy of each system for estimating fish lengths *in situ*. The SCs were positioned at BD1 during all reef site surveys because all three BDs provided length estimates that were below the accuracy threshold in the pool experiment, but BD2 and BD3 reduced ROV maneuverability in strong currents. The two GoPro Hero5 cameras mounted in stereo were set to the narrow field of view (49.1° vertical and 64.6° horizontal FOV, 28 mm focal length) with 1080-p resolution and 120-fps frame rate. Increased light intensity and much shorter video durations during field surveys enabled the use of higher frame rates reduces motion blur and enhances species identification during video processing in the laboratory.

The calibration checkerboard used in the pool experiment was attached to an aluminum pole and submerged alongside the research vessel to collect paired videos for calibrating SCs in the field. A small, handheld flashlight was triggered immediately prior to deploying the ROV to allow for video synchronization and image extraction during post-processing of videos in the laboratory. The ROV was then deployed just below the surface and several transects were flown perpendicular to the submerged checkerboard to enable extraction of paired images for calibration. Transects were flown by initially positioning the ROV perpendicular to the checkerboard at a distance of approximately 1 m. The ROV then was slowly flown in reverse until the checkerboard pattern was no longer clearly visible (i.e., >5 m). The ROV was then flown slowly towards the checkerboard until it filled the camera FOV (<1 m). This process was repeated three times to ensure at least 50 paired checkerboard images were available for the calibration algorithm. The calibration procedure took ≤5 minutes to complete.

A single calibration was used for all successive reef site surveys for the duration of each camera pair’s battery life (n = 5–8 reefs). Calibration videos were not collected prior to every survey because this would greatly reduce the number of sites sampled on a given day, and the rigid cases with secure mounting hardware provided reliably fixed camera positions during normal operations. An object of known length (603 or 364 mm PVC pipe, or 275 mm PVC disc) was submerged at each site and filmed, and later measured during video processing, at the end of each survey as a means to validate the SCs calibrated positions throughout each survey and through the duration of each pair’s battery life. A new calibration video was immediately collected following removal of cameras from their cases for battery replacement or to exchange memory cards.

Following SC calibration, the ROV was retrieved and the research vessel was positioned over a reef site. The flashlight was triggered onboard the vessel several times in simultaneous view of all three digital cameras immediately prior to each ROV survey. The ROV was then deployed to survey reef fish communities with a transect method at natural reefs, as described in Patterson et al. [44], and a point-count method adapted from Bohnsack and Bannerot [45] at artificial reefs, as described in Patterson et al. [38]. At natural reefs, four orthogonal 25-m long transects were flown from a central stationary point at a height of 1 m above the seafloor at a constant speed of ~1 kt. A weight (~7 kg) was attached to the ROV’s tether via a short (~2 m) rope 25 m from the ROV and deployed at the GPS coordinates, which provided the central point for each of four cardinal direction survey transects [44]. The GoPro Hero5 placed atop the ROV (center camera; Fig 1B) was positioned at a 45° downward angle from the horizontal axis to identify and enumerate reef fishes during transects, as well as to estimate fish lengths with the red laser scaler. The center camera was set to the wide FOV (94.4° vertical and 122.6° horizontal FOV, 14 mm focal length) at 2.7k resolution and 120-fps frame rate. At artificial reefs, the GoPro Hero5 was positioned at 0° angle from the horizontal axis to observe reef fishes during 360° spins conducted on opposite sides (1 m above the seafloor), atop (1 m above the top of the reef structure), and above (10 m above the top of the reef structure) each reef structure. During spins on opposite sides of each reef, the ROV was positioned such that the artificial reef module occupied 20% of the ROV’s FOV in real-time [38]. Regardless of reef type, one of the PVC objects described above was attached to the ROV’s tether such that the object was suspended 1 m above the seabed. At the end of each survey (~10 minutes in duration), the deployed PVC object was located along the tether and a single perpendicular transect (with the same methods described above for collecting video of the calibration checkerboard) was flown with the ROV to collect 10 paired images.

Calibration and survey videos were processed in the laboratory. The GoPro Hero5 provided high-resolution video for fish identification and enumeration during processing. In addition to collecting fish community data, the center camera was also used to estimate whether fish were appropriately oriented for collecting length measurements. Results of the pool experiment indicated that targets struck with both lasers simultaneously at an AOI ≤10° and a distance ≤5 m from the ROV could be measured with the RLS with a mean error within ±5%. The SCs with BD1 were capable of accurately measuring targets (mean error within ±5%) at an AOI ≤30° and a distance ≤5 m from the ROV, but we chose a conservative AOI of ≤25° to ensure that targets were oriented sufficiently for collecting accurate length measurements. Therefore, length was estimated for all reef fish that met the orientation criteria for either measurement system. Length was also estimated for the PVC object at each reef site and Eq 1 was utilized to compute the percent error in object length estimates as a means to validate the calibration file at each site.

## Results

### Pool experiment

**Fig 3:**
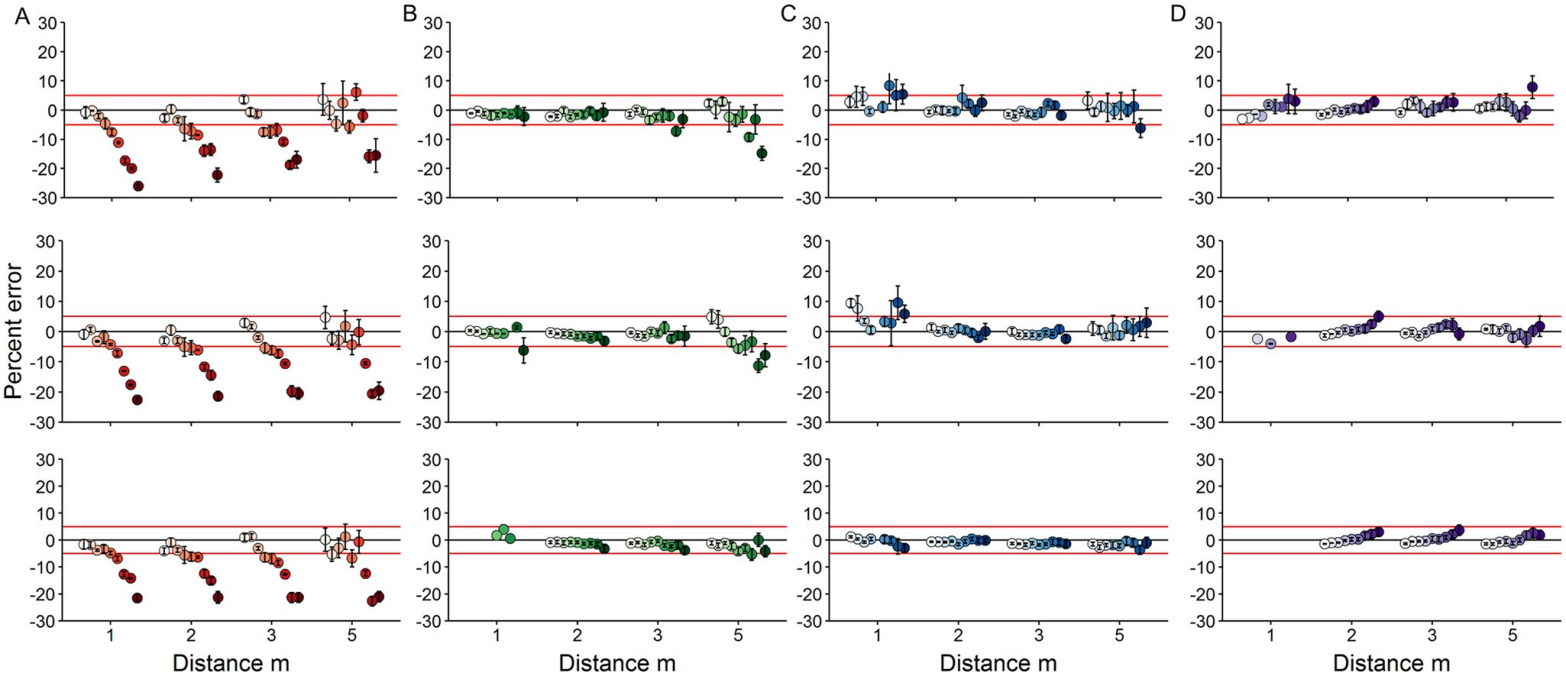
Pool experiment length estimate error plots. Mean percent error (±95% CIs) in red snapper fork length estimates with increasing distance (m) and angle of incidence (AOI, degrees) as measured with the A) RLS (75 mm baseline, red gradient), or SCs at baseline distances of B) 406 mm (BD1, green gradient), C) 610 mm (BD2, blue gradient), or D) 762 mm (BD3, purple gradient) in the pool experiment. Filled circles (n = 9) from left to right in each panel indicate AOIs from 0 to 40° at 5° increments. Horizontal red lines indicate the ±5% error thresholds. Top, middle, and bottom rows in each column indicate measurements for small (288 mm FL), medium (552 mm FL), or large (890 mm FL) paper red snapper models, respectively. Distance and AOIs with length errors below 5% were deemed viable for collecting fish length measurements from ROV survey videos in the field experiment.

Mean PE of target length estimates measured with the RLS was within the ±5% error threshold at AOIs ≤10° at all distances, and up to 30° at 5 m but with greater variability (Fig 3; S1 Data). Mean PE was also within the ±5% threshold for SCs at all three BDs for nearly all angles at all distances tested. At BD1 (Fig 3, column B), variability increased for measurements at the 5 m distance for small and medium fish models with increasing AOI. At BD2 (Fig 3, column C), variability in MPE estimates at 5 m increased slightly for small and medium targets at 5 m but was greater at 1 m with some AOIs exceeding +5%. At BD3 (Fig 3, column D), all mean PE estimates were within the error threshold at all distances and AOIs except when the small model was measured at 40° at 5 m. All estimates of the large model were within the ±5% error threshold, regardless of the BD. However, the large target could not be measured at 1 m distance for most AOIs with SCs at either BD1 or BD3 due to the narrow FOV. The 552 mm red snapper model also could not be viewed at several AOIs at 1 m distance with BD3. Bias was BD-specific, with BD1 having increasingly negative bias (underestimated lengths) with increasing AOI, BD2 having no consistent pattern, and BD3 having increasingly positive bias (overestimated lengths) with increasing AOI. Length estimates produced with the RLS showed a negative bias that increased in magnitude with AOI but not with distance.

**Fig 4:**
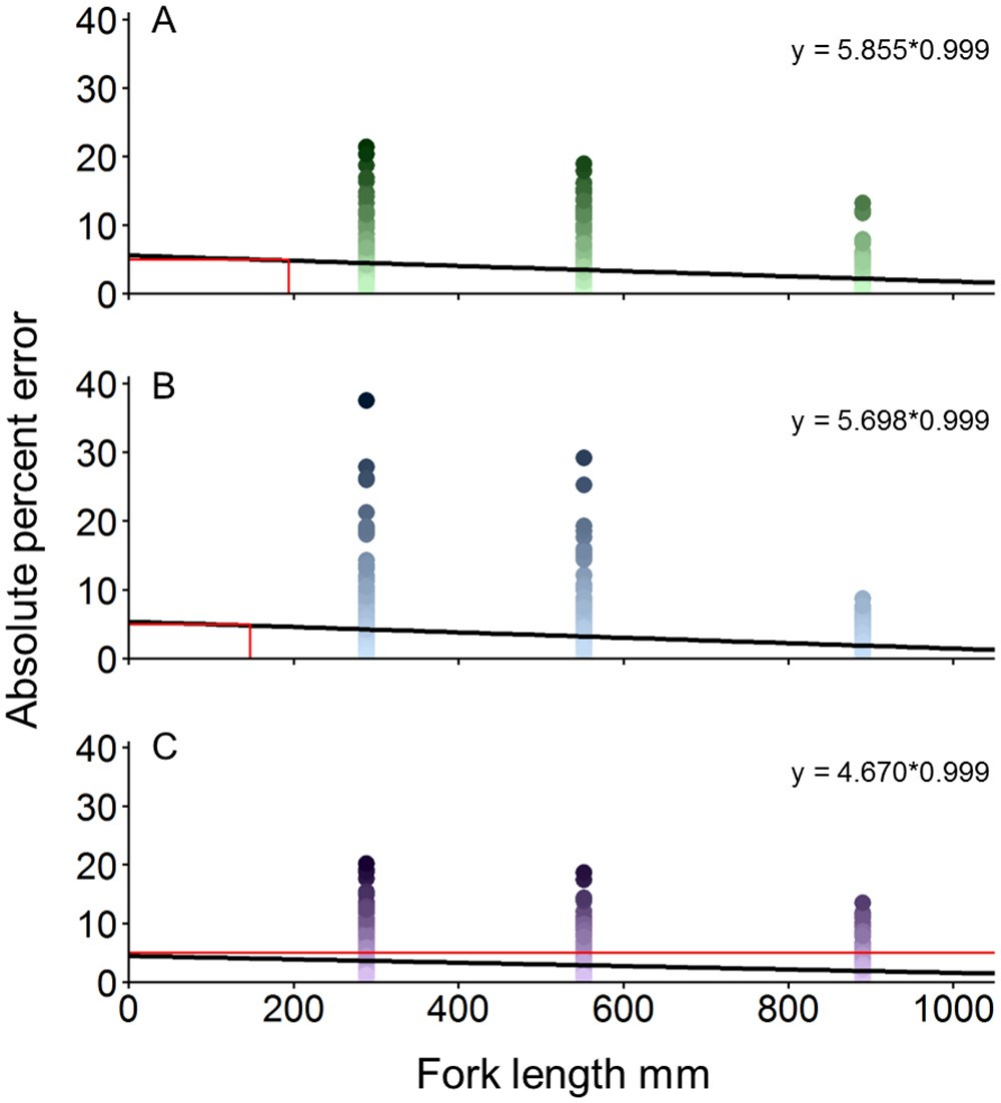
Pool experiment length estimate regression plots. Gamma GLM regressions of absolute percent error estimates versus model fork length for SCs at A) BD1 (406 mm), B) BD2 (610 mm), or C) BD3 (762 mm). Red lines indicate the length at which each regression intersects the *a priori* specified 5% error threshold, which occurs at 194 mm in A and 147 mm in B. All length estimates were below the error threshold in C.

Results of the Gamma GLM model for mean APE indicated that AOI was a significant main effect (p < 0.001) but that AOI interacted significantly with distance and the measurement system. Specifically, the BD1*Distance*AOI (coefficient = 0.015; p = 0.031) and BD3*Distance*AOI (coefficient = 0.015; p = 0.027) interactions indicated SC systems significantly decreased APE with increasing AOI by distance compared to estimates from the RLS (the base level); the p-value for the interaction term BD2*Distance*AOI was p = 0.066 (coefficient = 0.012). Mean APE for the small model was not significantly different from length estimates for medium (p = 0.980) or large (p = 0.653) models, nor was error significantly different when a fish model was one (p = 0.518) or two (p = 0.376) positions above or below the center view of the camera. Regressions of fish model FL versus APE with fitted data from the Gamma GLM for each SC BD indicated the minimum estimable fish model FL was 194 mm for SCs at BD1 and 147 mm at BD2; all model lengths were estimable at BD3 (Fig 4).

### In situ target estimates

**Fig 5:**
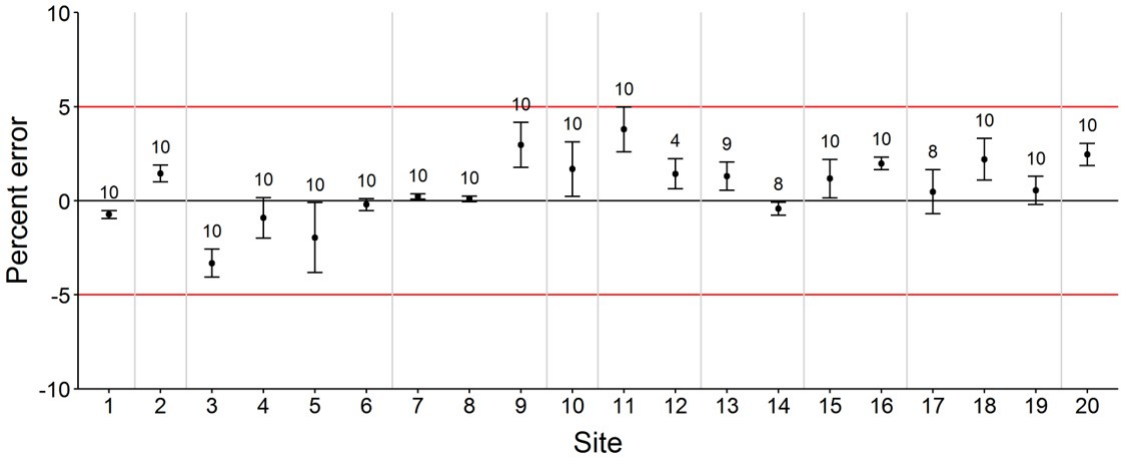
Field experiment length estimate error plot for objects of known length. Mean percent error estimated for objects of known length from paired still images obtained with SCs at BD1 (406 mm) mounted to an ROV deployed at reef sites (n = 20) in the northern Gulf of Mexico in 2018. Error bars indicate ±1 standard error of the mean. The number of observations is shown above each estimate. Horizontal red lines indicate the ±5% error threshold. Object length estimates for sites 1–4 and 20 were for a 603.3 mm PVC pipe, sites 5–10 were for the diameter of a PVC disc (274.6 mm dia), and sites 11–19 were for a 364.0 mm PVC pipe. Vertical gray lines bracket length measurements estimated with each set of calibration parameters.

**Fig 6:**
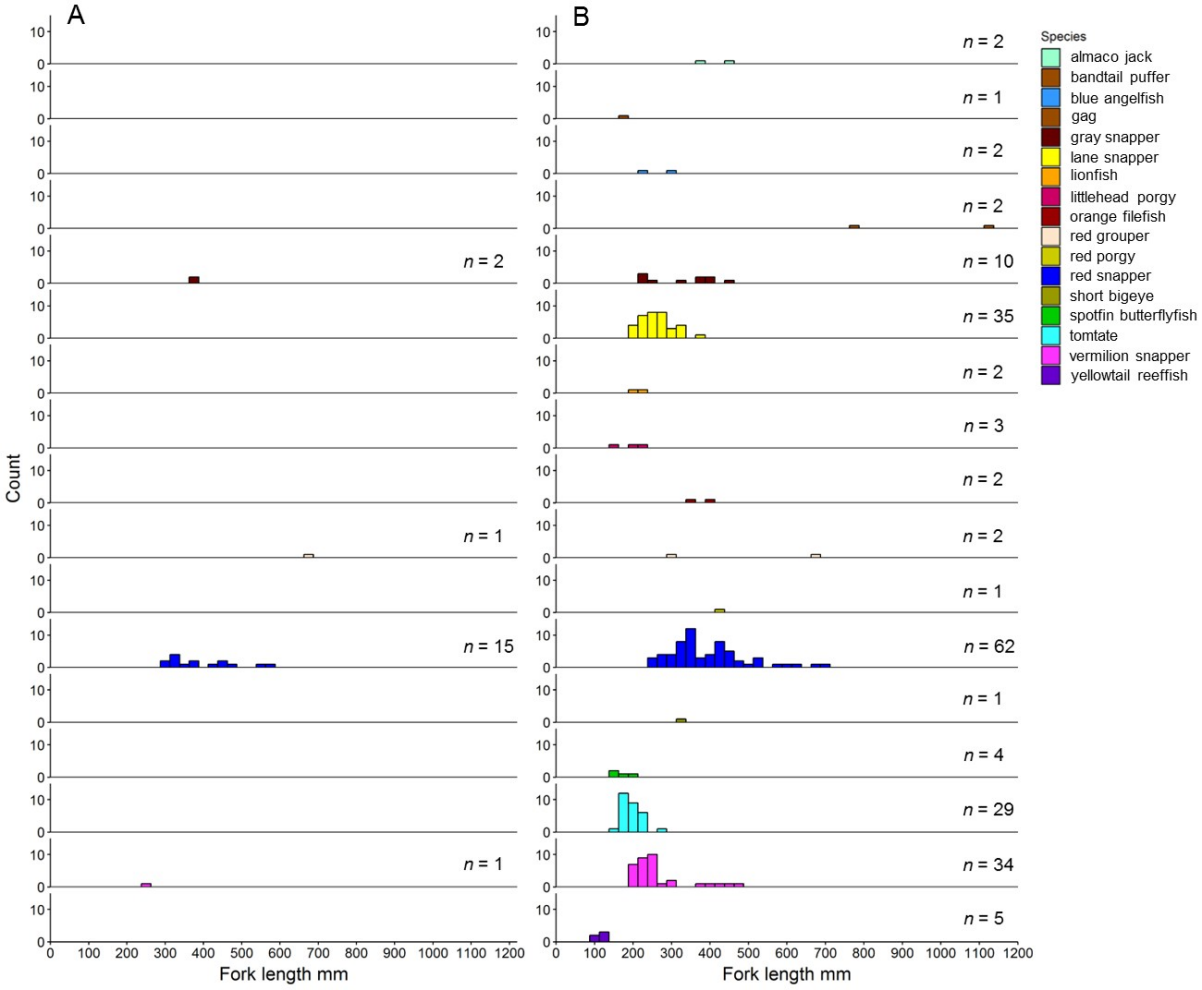
Field experiment frequency histograms. Frequency histograms (counts, 30 mm bins) of fork length estimates for 197 individuals among 17 species measured with a A) red laser scaler or B) stereo cameras with BD1 (406 mm) at reef sites (n = 20) surveyed in the northern Gulf of Mexico in 2018. The number of length estimates for each species is indicated on the right side of each panel.

Twenty reef sites were surveyed during field trials with the ROV integrated with the RLS and SC systems (See S1 Data for GPS locations). Survey sites consisted of unstructured hard bottom (n = 8), low-relief natural reef (n = 10), and artificial reefs (n = 2). PVC object lengths (*n* = 189) estimated at each of the 20 sites had a mean PE (±SE) of 0.76% (± 0.21%) among all sites, and at no site did mean PE exceed the ±5% threshold (Fig 5; S1 Data). In total, 3,249 individuals among 40 species were observed during ROV surveys (Table 1). An additional 175 individuals were unable to be identified due to small size, distance from camera, or visibility issues. Of the total number of individuals and species observed, 19 individuals among 4 species were scaled with the RLS, while 197 individuals among 17 species were measured with the BD1 SC system (Fig 6; S1 Data). All individuals scaled with the RLS also were observed and measured with the SCs; red snapper were the most frequently measured with either method.

**Table 1:** List of observed species and their composition.

Scientific name	Common name	Count	%Frequency
*Aluterus schoepfii*	orange filefish	2	<0.1
*Balistes capriscus*	gray triggerfish	4	<0.1
*Calamus proridens*	littlehead porgy	11	<0.1
*Caranx crysos*	blue runner	1	<0.1
*Carcharhinus obscurus*	dusky shark	2	<0.1
*Centropristis ocyurus*	bank seabass	1	<0.1
*Chaetodon aya*	bank butterflyfish	1	<0.1
*Chaetodon ocellatus*	spotfin butterflyfish	12	<0.1
*Chromis enchrysura*	yellowtail reeffish	33	<0.1
*Chromis scotti*	purple reeffish	1	<0.1
*Diplectrum formosum*	sand perch	6	<0.1
*Epinephelus itajara*	goliath grouper	1	<0.1
*Epinephelus morio*	red grouper	3	<0.1
*Equetus lanceolatus*	jackknife fish	1	<0.1
*Pareques umbrosus*	cubbyu	2	<0.1
*Haemulon aurolineatum*	tomtate	786	0.2
*Halichoeres bivittatus*	slippery dick	3	<0.1
*Halichoeres poeyi*	blackear wrasse	1	<0.1
*Holacanthus bermudensis*	blue angelfish	9	<0.1
*Lactophrys quadricornis*	scrawled cowfish	1	<0.1
*Lutjanus campechanus*	red snapper	261	0.1
*Lutjanus griseus*	gray snapper	28	<0.1
*Lutjanus synagris*	lane snapper	116	<0.1
*Mycteroperca microlepis*	gag	2	<0.1
*Mycteroperca phenax*	scamp	1	<0.1
*Ogocephalus radiatus*	polkadot batfish	1	<0.1
*Pagrus pagrus*	red porgy	9	<0.1
*Pristigenys alta*	short bigeye	3	<0.1
*Ptereleotris calliura*	blue dartfish	49	<0.1
*Pterois volitans*	red lionfish	24	<0.1
*Raja texana*	roundel skate	1	<0.1
*Rhinobatos lentiginosus*	Atlantic guitarfish	1	<0.1
*Rhomboplites aurorubens*	vermilion snapper	1822	0.6
*Rypticus maculatus*	whitespotted soapfish	3	<0.1
*Seriola dumerili*	greater amberjack	20	<0.1
*Seriola rivoliana*	almaco jack	14	<0.1
*Sphoeroides spengleri*	bandtail puffer	1	<0.1
*Stegastes leucostictus*	beaugregory	5	<0.1
*Synodus intermedius*	sand diver	1	<0.1
*Xyrichtys novacula*	pearly razorfish	6	<0.1

Number of observations and relative frequency of fishes observed during ROV video surveys at 20 reef sites in the northern Gulf of Mexico in 2018.

**Fig 7:**
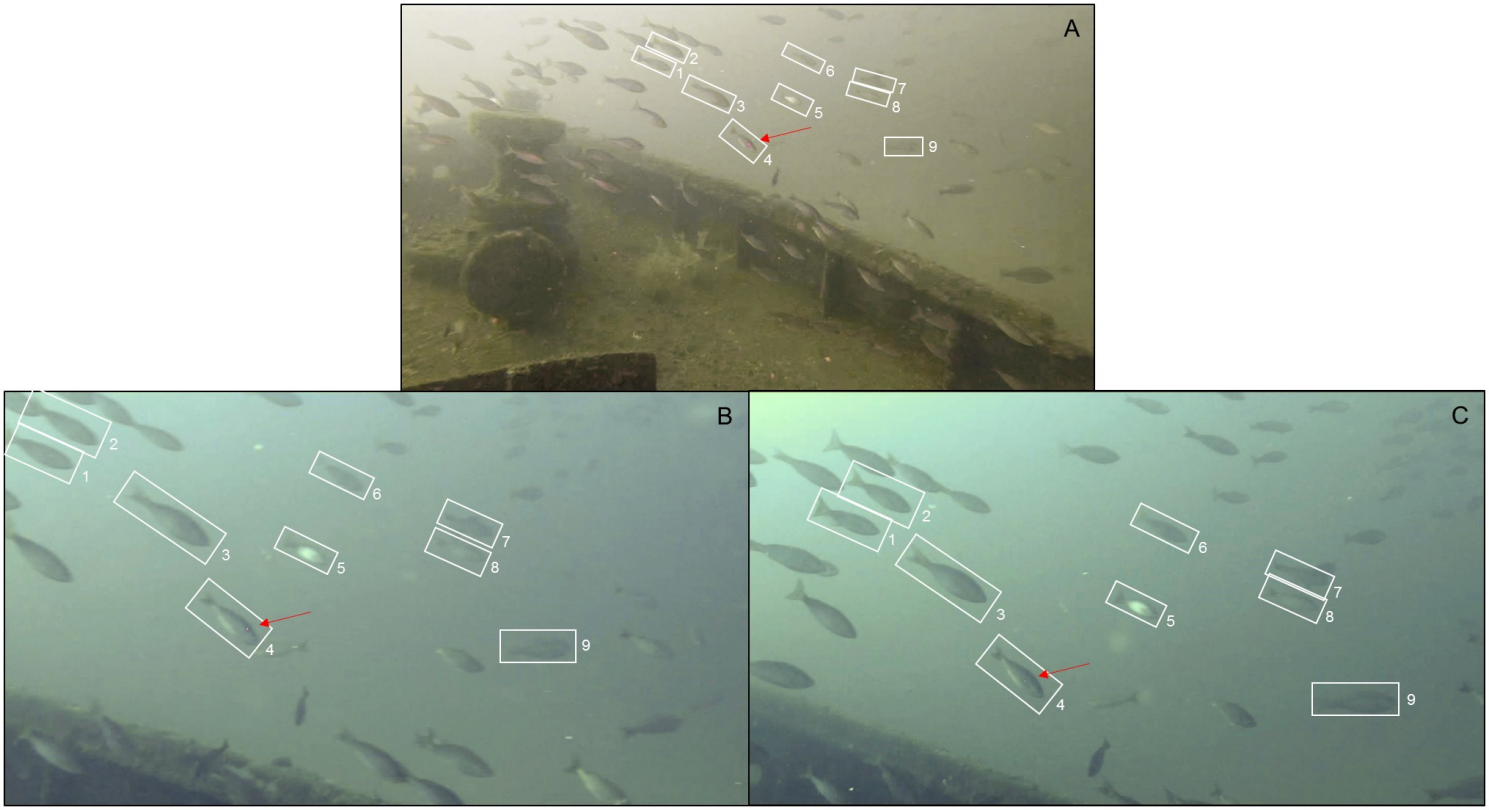
Example stereo camera vs red laser scaler sample availability. School of vermilion snapper passing in front of A) the center GoPro camera mounted atop the ROV, and the B) left and C) right stereo cameras during an artificial reef survey in the nGOM in 2018. Numbered white boxes indicate individuals whose lengths could be estimated with the SC system because they are entirely visible in both views simultaneously and are oriented ≤25° from perpendicular to the ROV’s longitudinal axis based on their orientation in the horizontal plane in panel A. A single laser point is visible on individual 4 in all three images as indicated by the red arrow, which is insufficient to estimate length with the RLS.

Red snapper mean fork length estimated with the SC system (393.1 ±12.8) was similar to the mean estimated with the RLS (394.4 ±22.0), but the SC system provided 47 additional length measurements compared to the RLS, including eight individuals <300 mm and four individuals >600 mm FL that could not be measured with the RLS because they were not struck simultaneously by both laser points at the correct orientation (≤10° from perpendicular to the ROV’s center axis). No red snapper <300 mm FL were scaled with the RLS and only one individual scaled exceeded 600 mm FL. The number of individual length measurements for species other than red snapper was 10x greater with the SC system than the RLS. Furthermore, four fishery species (i.e., gray snapper, *Lutjanus griseus*, lane snapper, *Lutjanus synagris*, tomtate, *Haemulon aurolineatum*, and vermilion snapper, *Rhomboplites aurorubens*) were measured with the SC system that were never or rarely scaled with the RLS (Fig 6). The SC system also produced length estimates for an additional 12 low-abundance species that were never measured with the RLS (Table 1; Fig 6). These low-abundance species were predominantly comprised of small individuals ≤300 mm, which were rarely struck by both laser points simultaneously due to a combination of small body size and orientation in the horizontal plane (see Fig 7 for example image). Larger individuals also are only infrequently scaled by lasers because the RLS requires they swim directly into the center-of-view.

## Discussion

Study results indicate that a SC system integrated with a (micro) mini ROV is a viable means to collect robust length estimates for a wide variety of reef fishes. Compared to an RLS, SCs substantially increased the number of reef fish length estimates by enabling measurements to be taken for fishes at greater AOIs from a much greater portion of the video field-of-view. In addition to reducing length estimation error, SCs also reduced overall bias in reef fish length estimates by allowing measurement of fishes shorter than the RLS baseline (75 mm), which greatly increased the number of estimates taken for relatively small fishes (<300 mm). Shorter SC BDs produced robust reef fish length estimates up to 30°, but the majority of reef fishes could be viewed at a variety of distances and angles, especially at 2 to 3 m where precision and accuracy were high for SCs at all three BDs tested in the pool experiment. Wider BDs may be necessary when a significan portion of target species occur at great distances (≥5 m), such as when studying highly mobile, shy, or rare species [24, 46, 47], species that exhibit avoidance behaviors [28, 47, 48], or relatively small individuals for which AOI is difficult to estimate reliably (e.g., Pomacentrids). The efficacy of stereo cameras for collecting robust length measurements have been demonstrated with a variety of stationary platforms [30], divers [49–52], and working-class ROVs [53], but studies utilizing SCs integrated with (micro) mini-class ROVs [28] to collect *in situ* length estimates via short BDs are limited [52].

Accuracy and precision estimates reported in our study are similar to results from previous works conducted in controlled enclosures with manually positioned cameras, which demonstrated the potential for small action cameras to collect highly accurate (<5% error) length measurements at a range of distances and AOIs [43, 49, 54, 55]. Greater BDs provide more contrast between paired images and allow greater accuracy and precision in length estimates at greater angles of incidence [40, 50, 54]. Shortis and Harvey [50] concluded that a camera separation of 1.4m was ideal for collecting fish measurements up to 5 m away (following a BD to target distance ratio of 3.6) based on frequently observed distances between divers and reef fishes. For BDs >700 mm, estimates are highly accurate (<5% error) at target distances ≥5 m and AOIs up to 40° while decreasing the BD decreases the distance and AOI available to collect viable length estimates [49, 54, 55]. Field experiments suggest that a smaller window of opportunity for collecting *in situ* length measurements is not problematic because mobile survey gears do not induce strong behavioral responses in many species and observe most individuals at a variety of distances and angles; baited gears draw in many carnivorous or scavenging species to relatively close distances from cameras [28, 46]. Although we did not include multiple calibration techniques or brands of video recording devices in our pool experiments, Boutros et al. [49] reported similarly high accuracy in target length estimates collected between 2D (checkerboard) and 3D (cube) calibration techniques [56, 57]. Letessier et al. [55] reported similar accuracy and precision to that reported in our study with earlier model GoPro cameras (Hero 2) and found no significant difference in length estimates between Sony and GoPro brand cameras between paired fish length estimates collected *in situ*.

Stereo cameras integrated with mini ROVs provide several advantages compared to other methods for collecting fish length estimates. Unlike larger survey gears, mini ROVs are easily deployed from small vessels by a single person, which facilitates lower operating costs. Compared to divers, ROVs can access greater depths (>300 m) without any cumulative restrictions on dive duration or health risk. Unlike baited stationary camera systems, quantitative survey methods with mini ROVs allow the collection of density estimates by estimating the area sampled [38, 44]. However, Schramm et al. [28] recommends that stationary baited cameras should complement mobile gears when estimates of diversity are desirable because baited systems are likely to observe significantly more species.

Integrating ROV-based methods with stereo cameras and StereoMorph video analysis software provides a means to fully eliminate the need for divers and pool facilities because the 2-dimensional checkerboard needed to calibrate the SC systems can be easily submerged and positioned from the side of a vessel via an extendable aluminum pole. Attaching the checkerboard to an extendable pole provides a rapid (<5 min) and easily deployed field method to obtain new calibration coefficients should any unexpected movement occur in the fixed position of the SC system. In our experience, the careful handling required when deploying expensive ROV equipment along with slow flying speeds underwater virtually eliminate potential collisions that could necessitate recalibration. Instead, recalibration is nearly always associated with camera battery replacement, which typically occurs only once or twice per day due to the relatively short survey times (<10 min) required for each site. Letessier et al. [55] reported that removing GoPro cameras multiple times per day from the standard underwater housing for battery replacement had no meaningful effect on accuracy or precision of *in situ* length estimates. We simply chose to recalibrate after battery replacement as a precautionary measure because the minimal time required to collect additional calibration videos is short (<5 min). We also utilized objects of known length to verify measurement accuracy in successive survey videos for each calibration to maximize our confidence in collecting consistently accurate length estimates. Small, lightweight, extended-life batteries (up to 24 hrs) with waterproof cases have recently become available for GoPro brand cameras that could reduce the number of daily calibrations to a single event to eliminate this potential source of error.

The checkerboard square size and corner number appropriate for successful SC calibrations depends upon the size of desired target observations and water conditions [40, 49]. Olsen and Westneat [40] recommend a checkerboard image at least 40 pixels wide with as many internal corners as possible to maximize calibration accuracy. More internal corners can increase calibration accuracy by providing more data points, particularly for correcting lens distortion. However, the checkerboard squares must also be large enough for corners to be detected at the expected target distances. Thus, smaller checkerboards may be better suited to calibrating measurements of smaller individuals at close range, while larger ones are more appropriate for larger individuals viewed at greater distances. Our checkerboard design was successfully detected in Stereomorph less often at distances of 5 m due to decreasing contrast between the black and white squares and interference from bubbles and drifting particles. Regardless, the overwhelming majority of fishes observed during ROV survey videos were between 1 and 5 m from the SC system.

The accuracy of length measurements collected with laser-based methods are ultimately limited by 1) the parallax effect, 2) laser separation distance, 3) size bias towards larger individuals, 4) the frequency of scaling events at appropriate AOIs, or 5) beam contact distortion [36, 38, 53, 58]. In a controlled pool experiment, Patterson et al. [38] estimated that an RLS with 100 mm baseline provided accurate (mean PE within ±5%) length estimates of fish models at distances ≤2.5 m and AOIs ≤15°. Our estimates of measurement accuracy likely differed from Patterson et al. [38] due to differences in video capture technology and laser spacing (100 versus 75 mm baselines). Patterson et al. [38] utilized a VideoRay Pro3 mini ROV and captured still images directly from the ROV’s internal camera, which had lower resolution than the digital GoPro Hero5 cameras utilized in our study.

With ROVs, especially mini-class ROVs, considerations that compete with image resolution and minimizing bias include payload, hydrodynamics, and maneuverability. The VideoRay Pro4 ROV produces 9.5 kg of thrust at a maximum speed of 2 m/s but can support only a relatively small payload without considerable reduction in hydrodynamic performance, especially when operating against a hydrodynamic current. Thus, the SC system, including waterproof cases and mounting bracket, could not greatly exceed the total ballast weight typically utilized with the ROV (1.5 kg) without necessitating additional specialized flotation. The camera and bracket design for the SC system with BD1 had a mass of only 0.7 kg, which enabled us to simply remove ROV ballast weights to offset that mass. The ROV’s flotation block can be adapted or additional flotation can be affixed to the sled to offset the increased weight of heavier cameras or sturdier cases designed to withstand greater pressures at increased depth but will likely reduce ROV maneuverability.

Regarding camera settings, we recommend setting GoPro cameras to the narrow FOV to minimize barrel distortions [43, 52, 54, 59]. When ambient light is sufficient, we recommend maximizing the video resolution and/or frame rates of cameras used in field surveys to increase placement accuracy of landmark points to maximize measurement accuracy during image digitization. In our pool experiment, we noticed pixilation effects at 5 m distance that decreased our ability to accurately place digital landmarks for estimating fish model lengths. The importance of maximizing accuracy and precision is inversely related to fish size because a relatively small absolute errors will result in disproportionately greater percent errors when measuring smaller individuals. Higher frame rates can reduce motion blur as mobile fish swim through view, but inadequate lighting or rolling shutters can distort image quality of fast moving objects [60]. Higher resolution or frame rate settings will decrease camera battery life, which will necessitate more frequent recalibration. Ultimately, the capabilities of the ROV and specific research goals of the study will determine the appropriate SC design.

## Conclusions

Our results clearly demonstrate the efficacy of SC systems integrated with (micro) mini ROVs for estimating length distributions of reef fish communities. The specific design used (e.g., camera models, video resolution, and BD) must be considered against the size and behavior of the species of interest, the hydrodynamic capabilities of the ROV, and goals of the study. We recommend using the greatest BD possible to maximize measurement accuracy and precision, but all three BDs we tested produced accurate fish length estimates over most distances and target AOIs encountered during field surveys of fish communities commonly observed at northern GOM reef sites. We were successful in calibrating SC systems using a checkerboard deployed from the side of a vessel, but weather conditions, turbidity, and fine-scale visual obstructions can increase the duration of video necessary to extract a sufficient number (~50) of checkerboard image pairs for successful camera calibration. Thus, the checkerboard dimensions and deployment methods should be considered for the expected sea conditions and vessel design used for collecting calibration videos. Regardless of the region or application of interest, integrating SC systems with stationary gear or mobile platforms like ROVs enables much greater length composition data to be collected than with an RLS. Video surveys that collect only relative abundance estimates can be coupled with SC systems to also provide length composition estimates for input into stock assessments as well as hypothesis tests in ecological studies of length-dependent factors.

## Supporting information

S1 Data Complete set of data and metadata underlying all reported findings. (XLSX)
